# Efficacy of an Extract of *Ocimum tenuiflorum* (OciBest) in the Management of General Stress: A Double-Blind, Placebo-Controlled Study

**DOI:** 10.1155/2012/894509

**Published:** 2011-10-03

**Authors:** Ram Chandra Saxena, Rakesh Singh, Parveen Kumar, Mahendra P. Singh Negi, Vinod S. Saxena, Periasamy Geetharani, Joseph Joshua Allan, Kudiganti Venkateshwarlu

**Affiliations:** ^1^Clinical Pharmacology, OP Chaudhry Hospital and Research Centre, Lucknow 226 015, India; ^2^General Medicine and Cardiology, Lucknow 226 001, India; ^3^General Medicine, Bhatnagar Nursing Home, Lucknow 226 012, India; ^4^Statistics Centre, Institute for Data Computing and Training, Lucknow 226 012, India; ^5^Sannidhi, Gurgaon, India; ^6^Research and Development Centre, Natural Remedies, Karnataka, Bangalore 560 100, India

## Abstract

A randomized, double-blind, placebo-controlled study was conducted to evaluate the efficacy of OciBest, an extract of *Ocimum tenuiflorum* Linn. in symptomatic control of general stress. The participants received either placebo (*n* = 79) or OciBest (*n* = 71; 1200 mg of actives per day) for six weeks. The severity of stress-related symptoms was self-evaluated by patients at weeks 0, 2, 4 and 6 of the trial period using a symptom rating scale. After six weeks of intervention, scores of symptoms such as forgetfulness, sexual problems of recent origin, frequent feeling of exhaustion, and frequent sleep problems of recent origin decreased significantly (*P* ≤ 0.05) in OciBest group as compared with placebo group. Also, the total symptom scores of OciBest group revealed significant reduction (*P* ≤ 0.05) as compared to placebo group. The overall improvement in OciBest group was found to be 1.6 times or 39% more in the control of general stress symptoms with respect to placebo. No adverse events were reported during the study. The findings revealed that OciBest was found to be effective and well tolerated by all the patients over the six weeks of study period.

## 1. Introduction

In modern society, stress is a common problem faced by everyone in day-to-day life from different sources like job, family problems, pollution, noise etc. Stress is defined as psychological, physiological, and behavioural response by individuals when they perceive a lack of equilibrium between the demands placed upon them and their ability to meet those demands [1]. The level of stress depends on the threshold of each individual. Over a period of time the response to stress aggravates and leads to ill-health [1, 2]. 

The common symptoms of stress include fatigue, tenseness, irritability, apathy, sleeping disorders, emotional instability, thoughts, and concentration problems [3]. Present generation people are more ravaged by the consequences of stress than at any time in past, and financial expenses in terms of long-term sick leave due to stress-related mental disorders are extensive [4]. Nearly 75% to 90% patient visits to primary care physicians are related to stress problems. In USA, health and efficiency costs of worker stress were reported to be between $50 and $150 billion per annum, and irrepressible stress revealed to be high risk factor for cancer and heart disease than either cigarette smoking or intake of high-cholesterol diets [5–8]. Psychological problems due to enormous workloads among employees have increased at a faster rate in many countries. In England, around 40 million workdays per year have been lost owing to mental and emotional problems [9]. 

Management of any unusual stress has acquired significant implications in daily life. Whereas complete avoidance of stress is unlikely, any intervention that facilitates elevation of threshold level can be beneficial. In order to curtail the economic losses towards the stress-related disorders and to improve the quality of life, traditional therapies seem to be promising alternatives. Indian system of traditional medicine describes remedies based on herbal supplementation, minerals, and other therapeutic procedures for enhancing physical and mental performance to evade the stress levels [10–13].

*Ocimum tenuiflorum* Linn. (synonym: *Ocimum sanctum*), generally known as Tulsi or Holy Basil, belongs to family lamiaceae. In Ayurveda, *O. tenuiflorum* has been used for adaptogenic/antistress activity [14]. It has been widely reported to possess antipyretic [15], antiasthmatic [16], antioxidant [17, 18], analgesic [19], and anti-inflammatory properties [20]. *O. sanctum* prevented reduction in the levels of brain catecholamine and monoamine oxidase and increase in dopamine and 5-hydroxytryptamine in rats exposed to swimming and gravitational stress [21].

Despite the availability of extensive preclinical evidences on antistress activity of *O. tenuiflorum*, authenticated clinical data are found to be lacking. A study by Bhattacharyya et al. that revealed the promising effects of *O. sanctum* (500 mg/capsule, twice daily after meal) for a period of two months in the management of patients suffering from generalized anxiety disorder (GAD) remains the only pertinent clinical evidence published on the medicinal herb till date [22].

## 2. Participants and Methods

### 2.1. Participants

The present study was carried out at two different centers in Lucknow, India between April 2008 and September 2009. Patients were recruited according to the inclusion criteria if they (i) were aged between 18–65 years, (ii) had given written consent, (iii) agreed to come for followup at week 2, 4, and 6 irrespective of any relief or appearance of side effects, and (iv) were suffering from at least any three of the symptoms of stress shown (Table 1). Patients were excluded if they (i) were unable to give voluntary consent, (ii) had history of significant cardiac, hepatic, renal, brain, or blood-allergic diseases, (iii) had physical disabilities, (iv) were taking allopathic or herbal medicines or participating in any other clinical trial, or (v) were pregnant/breast feeding.

One sixty-seven patients were screened for selection criteria, of which 158 were selected for the study after obtaining the written consent from each individual by the investigators. The study was approved by Institutional Ethics Committee.

### 2.2. Study Intervention

OciBest, an extract of whole plant of *O. tenuiflorum* Linn. was developed by M/s Natural Remedies Pvt. Ltd., Bangalore, India. The extract was ensured to comply with phytochemical specifications, namely, Ociglycoside-I (Hydroxychavicol glucoside/4-allyl-1-O-*β*-D-glucopyronosyl-2-hydroxybenzene; >0.1% w/w), rosmarinic acid (>0.2% w/w), and triterpene acids (>2.5% w/w). The composition adhered to the international quality requirements which included analysis of solvent residues, heavy metals, pesticide residues, and microbial contamination. The placebo capsules contained microcrystalline cellulose. Each OciBest capsule contained 400 mg of actives. The placebo and OciBest were filled in “0” size blue-coloured, hard-gelatin capsules that could not be distinguished from each other. Each container was packed with 42 capsules and labeled with code number.

### 2.3. Randomization and Blinding

The patients enrolled for clinical trial were allotted to placebo and OciBest groups. To identify the patient code, a list of unique integer random numbers were obtained after generating the random allocation number using a computer-aided random series programme. According to the random allocation sequence, the distinct random numbers were mentioned in the respective containers (placebo or OciBest). The packed containers were dispatched from Natural Remedies Pvt. Ltd., Bangalore, India to the study centers. The entire process was accomplished in concealed manner. As per enrollment and random allocation sequence the pharmacist dispensed the study intervention and was responsible for the same. The study investigators, pharmacist, and the participants were all blinded during the trial period.

### 2.4. Study Protocol

The study was performed in a randomized, double-blind, placebo-controlled manner. One fifty-eight patients were selected for the study and randomly assigned to placebo and OciBest group. Each patient was handed over one container and advised to take one capsule after breakfast and two capsules after dinner (total 1200 mg of actives per day) for 2 weeks. After 2 weeks, the patient was asked to report for followup and symptoms reassessment. Any unconsumed capsules were counted and recorded for compliance. Fresh container of 42 capsules was provided after recording the compliance. Similar process was repeated when the patient reported for followup during week 4.

### 2.5. Outcome Measures

The symptom scores of patients (Table 1) were assessed on week 0 and thereafter followup on weeks 2, 4, and 6 of the study period. On each occasion, the patients were asked to grade themselves for all individual symptoms based on the symptom rating scale [23].

### 2.6. Sample Size

The required sample size for difference between two means, that is, for a two sample *t*-test was estimated according to Dell et al. [24]. The sample size was calculated with a power of 90% and value of alpha = 0.05. Thus, minimum 65 subjects were required for each group and total 130 subjects for the whole study. Due to self-limiting condition in stress and as drop outs are common in clinical trial [25], additional subjects were added in each group.

### 2.7. Statistical Analysis

One hundred and fifty participants were considered for statistical analysis. The baseline demographic characteristics of two groups were compared by independent Student's *t*-test. The score of each symptom was analyzed using RMANOVA. The statistical significance was set at *P* ≤ 0.05. The statistical applications were performed using SPSS (version 13) and STATISTICA (version 7.0) softwares. The effect of two groups on each symptom and total symptoms scores were calculated as the reduction in symptom severity scores from baseline (week 0) to final assessment (week 6). The effect size of individual and total symptoms was calculated as the difference between the effects of placebo and OciBest. The overall effect size (%) between two groups was calculated as

(1) $$\text{Overall}\;\text{effect}\;\text{size}\;\left(\text{\%}\right)=\left[\frac{\text{Ocibest}-\text{Placebo}}{\text{Ocibest}}\right]\times 100.$$

## 3. Results

### 3.1. Demographic Data

Out of 167 subjects assessed for eligibility, a total of 158 patients (placebo = 82; 45 males and 37 females and OciBest = 76; 44 males and 32 females) who fulfilled the selection criteria and willing to give informed consent were enrolled in the study and randomized to either placebo or OciBest groups. On comparison, the demographic characteristics of all patients at baseline did not differ significantly in any of the parameters (Table 2).

### 3.2. Symptomatic Assessment of Efficacy

The symptom scores of patients of placebo and OciBest groups were summarized in Table 3. There was no significant difference between the individual symptom scores of participants of OciBest group as compared to placebo group on week 0. In both the groups, mean scores of all individual symptoms showed a decreasing trend from week 0 to week 6 except for few symptoms in placebo which either remained constant (Avoidance of even familiar people, and missing appointments) or got aggravated (Blurring of vision, abnormal sensory perceptions, and frequent sleep problems) from week 4 to week 6.

Mean scores of all symptoms in OciBest group decreased significantly (*P* ≤ 0.05) from week 0 to weeks 2, 4, and 6 except for sexual problems of recent origin at week 2 while placebo group showed either nonsignificant decrease (abnormal perception of hearing, sexual problems of recent origin, abnormal sensory perceptions, avoidance of even familiar people, and missing appointments) or significant decrease from week 0 to weeks 2, 4, and 6 in few symptoms (headache, palpitation at rest, frequent gastrointestinal (GI) symptoms, frequent feeling of exhaustion, and frequent sleep problems) and week 0 to weeks 4 and 6 in other symptoms (Blurring of vision, forgetfulness, and quarrelsome behaviour). Significant decrease was noticed in abnormal movements only from week 0 to week 6 in placebo-treated group.

Among all parameters, mean scores of headache, palpitation at rest, frequent GI symptoms, and frequent sleep problems decreased significantly (*P* ≤ 0.05) from week 2 to weeks 4 and 6, and mean scores of forgetfulness, sexual problems of recent origin, quarrelsome behaviour and frequent feeling of exhaustion decreased significantly (*P* ≤ 0.05) from week 2 to week 6 in OciBest group. But in placebo group, most of the symptoms showed nonsignificant decrease except in few symptoms significant decrease (*P* ≤ 0.05) was noticed from week 2 to weeks 4 and 6 (frequent GI symptoms, frequent feeling of exhaustion and frequent sleep problems) and week 2 to week 6 (headache and quarrelsome behaviour).

On comparison, mean scores of forgetfulness, sexual problems of recent origin, quarrelsome behaviour, frequent feeling of exhaustion and frequent sleep problems decreased significantly (*P* ≤ 0.05) from week 4 to week 6 in OciBest group while in placebo group, all symptoms showed nonsignificant decrease from week 4 to week 6 except for quarrelsome behaviour. 

Comparing mean between both groups, the effect of both the treatments on all symptoms at week 2 and 4 was found to be the same while at week 6, symptoms of forgetfulness, sexual problems of recent origin, frequent feeling of exhaustion, and frequent sleep problems improved significantly (*P* ≤ 0.05) in OciBest-treated group as compared to placebo group. 

Total scores of all symptoms in both the groups decreased significantly (*P* ≤ 0.05) at all consecutive week intervals as compared to weeks 0, 2 and 4. At the end of the study, the between groups analysis of total symptom scores of OciBest revealed significant reduction (*P* ≤ 0.05) as compared to placebo. The effect of OciBest over placebo on comparison was considerable for all stress parameters. The overall effect size of OciBest was found to be 39% (1.6 times) higher than placebo (Table 3).

### 3.3. Drop Outs

**Figure 1:**
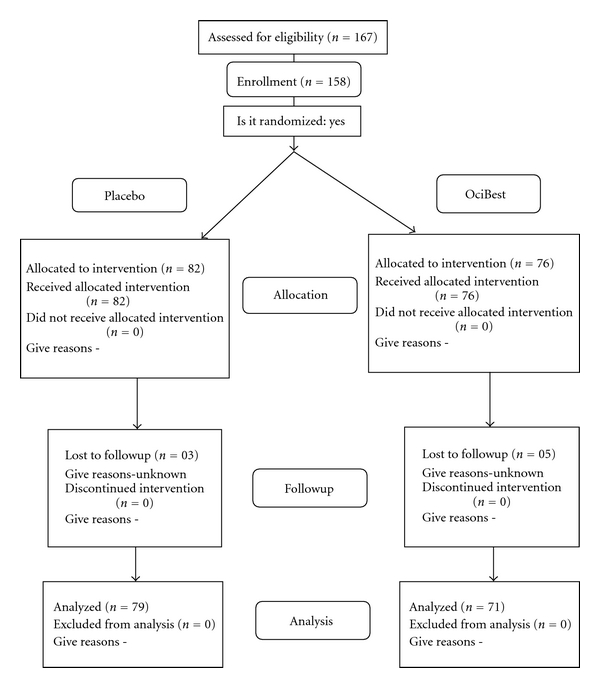
Disposition of patients.

Out of 158 enrolled patients at baseline, three patients in placebo and five patients in OciBest treated group did not turn up for followup and were excluded from data analysis. Thus symptom scores of 79 patients in placebo and 71 in test group were analyzed statistically (Figure 1).

### 3.4. Adverse Effects

None of the patients from both groups (placebo and OciBest) reported any adverse effects.

## 4. Discussion

Current pharmacological investigations focus on alleviating symptoms of stress since physical strains and psychological pressures have become integral and inevitable components of present life situations. Recently, herbal extracts and supplements have gained global recognition for stress-relieving potentials [26–28], in addition to popular medicinal uses and widely accepted safety profile [29–32]. 

The findings of a recent study proposed that people suffering from uncontrolled stress experienced impairment of short-term memory and other relevant functions in the prefrontal cortex induced by protein kinase C [33]. In the current study, supplementation with OciBest significantly decreased the intensity of forgetfulness to about one-third observed in placebo group. Additionally, the effect size was found to be the maximum (0.70) which elucidated that the participants of OciBest group were highly improved from forgetfulness than from any other symptoms.

Sexual problems and stress are related in several ways. Psychological causes that arise due to work-related stress result in sexual problems. Excessive stress can lead to reduced libido/sex drive in both sexes [34, 35]. Our findings showed that *O. tenuiflorum* extract remarkably reduced the symptom scores of sexual problems by 87.5% in comparison to patients of placebo group. 

In contrast to acute stress that accelerates neurotransmitters and hormones from the nervous and endocrine systems which in turn will enhance immune system functions, chronic stress aggravates exhaustion, distress, and disease [36, 37]. In the present investigation, OciBest administration provided effective relief from frequent feeling of fatigue. Similarly, disturbances in regular sleeping habits are encountered with stressful life style. Also, daytime sleepiness has been reported with prolonged stress exposures [38]. The results of the present clinical trial exhibited that the herbal intervention considerably reduced the intensity of symptom from 0.84 ± 0.14 to 0.27 ± 0.08. 

The overall effects of extract of *O. tenuiflorum* in patients with stress were found to be significant as compared to placebo which can be attributed to the influence of herbal supplement on four major symptoms like forgetfulness, sexual problems of recent origin, frequent feeling of exhaustion, and frequent sleep problems. The probable mechanisms of adaptogenic effects, from previously established studies, could be ascribed to neuroprotective [39], immunostimulant [40], free radical scavenging [41], nonspecific resistance inducing and plasma cortisol lowering [42] effects of *O. sanctum*.

The major phytochemical principles of Holy Basil largely contribute to its pharmacological actions. Saravanan and Pugalendi in 2006 reported that the triterpenoid ursolic acid at 20 mg per kg per day for 30 days could be able to ameliorate the oxidative stress in rat heart induced by chronic ethanol intoxication by reversing the peroxidative damages and concurrently enhancing the activities of antioxidant enzymes [43]. Another triterpenoid—Oleanolic acid protected mouse macrophages against oxidative and electrophile stress [39].

From the results of the present study, it is also evident that the severity of other symptoms such as headache, palpitation at rest, abnormal perception of hearing, blurring of vision, frequent GI symptoms, abnormal sensory perceptions, quarrelsome behavior, and avoidance of familiar people lessened to certain extent due to dietary supplementation. Although the scores of symptoms like abnormal movements of upper limb, tics, tremors, scratching, and missing appointments did not improve after six weeks of intervention, these symptoms are infrequently reported in stress patients. The positive trends of the present investigation can be corroborated with the research findings on *O. tenuiflorum* for stress management [44]. The different components of stress response include enhancement of central nervous system processes and the interaction between hypothalamus-pituitary-adrenocortical (HPA) axis [45]. Bhattacharyya et al. stated that, in human subjects, *O. sanctum* acts potentially in the regulation of HPA axis in stress-related disorders [22].

On the other hand, analysis of study results also indicated presence of placebo effect to some extent which is commonly anticipated as a regular incidence in several clinical trials [26, 46]. However, the critical observations on self-evaluated scores of study endpoints reinforce the fact that few symptoms in placebo group either remained constant (avoidance of even familiar people and missing appointments) or got aggravated (blurring of vision, abnormal sensory perceptions, and frequent sleep problems) from week 4 to week 6. 

Pertinent to safety, OciBest administration was found to be well tolerated in stress patients and no adverse events were recorded during the clinical trial. Earlier research by Bhattacharyya et al. in GAD patients also confirmed the overall safety of *O. sanctum* [22]. As mentioned previously, the wide margin of safety reported in experimental animal species tends to support the medicinal plant for human use [11].

In conclusion, the study findings revealed that OciBest, the whole plant extract of *O. tenuiflorum*, was found to be 1.6 times or 39% more effective in the management of stress symptoms in comparison to placebo group and the herbal supplementation was well tolerated by all the patients over the six weeks of study period.

## Figures and Tables

**Table 1:** Symptoms of stress.

(i) Headache	
(ii) Palpitation at rest	
(iii) Abnormal perception of hearing	
(iv) Blurring of vision	
(v) Forgetfulness	
(vi) Sexual problems of recent origin	
(vii) Frequent GI symptoms, lack of appetite, or dislike of even favourite food	
(viii) Abnormal movements of upper limb, tics, tremors, scratching	
(ix) Abnormal sensory perceptions particularly of lower limbs and face	
(x) Quarrelsome behaviour with later realizing of mistake	
(xi) Frequent feeling of exhaustion or overworked	
(xii) Frequent sleep problems of recent origin	
(xiii) Avoidance of even familiar people	
(xiv) Missing appointments for other things less important	

**Table 2:** Summary of demographic information.

Characteristics	Placebo	OciBest	*t*-value
Male/female	45/37	44/32	—
Age (years)	47.56 ± 1.11	48.53 ± 1.16	0.60^ns^
Weight (kg)	64.77 ± 1.19	64.30 ± 1.16	0.28^ns^
Height (cm)	161.07 ± 0.92	163.13 ± 1.20	1.37^ns^
Heart rate/min	79.20 ± 0.77	81.58 ± 1.11	1.79^ns^
BP diastolic (mmHg)	134.88 ± 1.87	133.28 ± 1.85	0.61^ns^
BP systolic (mmHg)	83.99 ± 0.85	82.58 ± 0.87	1.16^ns^

Values are expressed as mean ± SEM; Placebo *n* = 82; OciBest *n* = 76

Ns—non significant.

**Table 3:** Effect of OciBest on symptom scores of stress.

Symptoms	Groups	Assessment period				Effect^X^	Effect size^Y^
		Week 0	Week 2	Week 4	Week 6		
Headache	Placebo	1.34 ± 0.17	0.96 ± 0.14^a^	0.78 ± 0.13^a^	0.58 ± 0.12^ab^	0.76 ± 0.16	
	OciBest	1.24 ± 0.18	0.77 ± 0.14^a^	0.51 ± 0.11^ab^	0.31 ± 0.09^ab^	0.93 ± 0.15	0.17
Palpitation at rest	Placebo	1.29 ± 0.16	0.76 ± 0.12^a^	0.66 ± 0.11^a^	0.51 ± 0.11^a^	0.78 ± 0.15	
	OciBest	1.56 ± 0.19	0.93 ± 0.14^a^	0.59 ± 0.12^ab^	0.46 ± 0.11^ab^	1.10 ± 0.17	0.31
Abnormal perception of hearing	Placebo	0.25 ± 0.09	0.20 ± 0.08	0.18 ± 0.06	0.14 ± 0.06	0.11 ± 0.05	
	OciBest	0.45 ± 0.13	0.30 ± 0.09^a^	0.27 ± 0.09^a^	0.21 ± 0.08^a^	0.24 ± 0.08	0.13
Blurring of vision	Placebo	0.51 ± 0.13	0.37 ± 0.10	0.28 ± 0.08^a^	0.29 ± 0.09^a^	0.22 ± 0.07	
	OciBest	0.75 ± 0.14	0.45 ± 0.10^a^	0.31 ± 0.09^a^	0.28 ± 0.09^a^	0.46 ± 0.11	0.25
Forgetfulness	Placebo	1.25 ± 0.17	1.10 ± 0.16	0.99 ± 0.15^a^	0.95 ± 0.15^a^	0.30 ± 0.08	
	OciBest	1.32 ± 0.19	1.03 ± 0.16^a^	0.87 ± 0.14^a^	0.32 ± 0.08^abc^*	1.00 ± 0.15	0.70
Sexual problems of recent origin	Placebo	0.71 ± 0.16	0.65 ± 0.14	0.61 ± 0.14	0.56 ± 0.13	0.15 ± 0.05	
	OciBest	0.85 ± 0.17	0.75 ± 0.16	0.54 ± 0.12^a^	0.07 ± 0.03^abc^*	0.77 ± 0.16	0.62
Frequent GI symptoms	Placebo	1.61 ± 0.18	1.11 ± 0.14^a^	0.77 ± 0.13^ab^	0.59 ± 0.11^ab^	1.01 ± 0.15	
	OciBest	1.63 ± 0.20	1.07 ± 0.15^a^	0.63 ± 0.12^ab^	0.44 ± 0.10^ab^	1.20 ± 0.17	0.18
Abnormal movements	Placebo	0.39 ± 0.11	0.35 ± 0.11	0.28 ± 0.09	0.20 ± 0.08^a^	0.19 ± 0.08	
	OciBest	0.44 ± 0.12	0.27 ± 0.10^a^	0.23 ± 0.09^a^	0.20 ± 0.08^a^	0.24 ± 0.08	0.05
Abnormal sensory perceptions	Placebo	0.25 ± 0.09	0.16 ± 0.06	0.14 ± 0.06	0.15 ± 0.07	0.10 ± 0.05	
	OciBest	0.48 ± 0.13	0.32 ± 0.11^a^	0.27 ± 0.10^a^	0.25 ± 0.10^a^	0.23 ± 0.09	0.12
Quarrelsome behavior	Placebo	1.06 ± 0.17	0.96 ± 0.16	0.82 ± 0.15^a^	0.65 ± 0.12^abc^	0.42 ± 0.09	
	OciBest	0.90 ± 0.19	0.70 ± 0.15^a^	0.59 ± 0.13^a^	0.34 ± 0.09^abc^	0.56 ± 0.13	0.15
Frequent feeling of exhaustion	Placebo	2.00 ± 0.17	1.53 ± 0.14^a^	1.19 ± 0.13^ab^	1.04 ± 0.14^ab^	0.96 ± 0.14	
	OciBest	1.72 ± 0.19	1.13 ± 0.15^a^	0.86 ± 0.13^a^	0.37 ± 0.08^abc^*	1.35 ± 0.17	0.39
Frequent sleep problems	Placebo	1.49 ± 0.17	1.14 ± 0.14^a^	0.82 ± 0.13^ab^	0.84 ± 0.14^ab^	0.66 ± 0.12	
	OciBest	1.30 ± 0.19	0.89 ± 0.15^a^	0.58 ± 0.13^ab^	0.27 ± 0.08^abc^*	1.03 ± 0.17	0.37
Avoidance of even familiar people	Placebo	0.28 ± 0.10	0.23 ± 0.08	0.20 ± 0.08	0.20 ± 0.08	0.08 ± 0.06	
	OciBest	0.38 ± 0.12	0.21 ± 0.09^a^	0.17 ± 0.07^a^	0.10 ± 0.05^a^	0.28 ± 0.11	0.21
Missing appointments	Placebo	0.14 ± 0.07	0.13 ± 0.06	0.05 ± 0.03	0.05 ± 0.03	0.09 ± 0.05	
	OciBest	0.25 ± 0.09	0.14 ± 0.07^a^	0.11 ± 0.06^a^	0.07 ± 0.04^a^	0.18 ± 0.07	0.09
Total	Placebo	12.58 ± 0.48	9.66 ± 0.44^a^	7.77 ± 0.46^ab^	6.75 ± 0.45^abc^	5.84 ± 0.54	
	OciBest	13.27 ± 0.46	8.96 ± 0.43^a^	6.52 ± 0.45^ab^	3.69 ± 0.37^abc^*	9.58 ± 0.52	3.74

Values are expressed as mean ± SEM; Placebo *n* = 79; OciBest *n* = 71.

^a^ *P* ≤ 0.05 versus week 0; ^b^ *P* ≤ 0.05 versus week 2; ^c^ *P* ≤ 0.05 versus week 4.

*P ≤ 0.05— placebo versus OciBest.

^X^Difference in mean scores between week 0 and week 6; ^Y^D ifference in mean scores between placebo and OciBest.
